# Medical News and Miscellany

**Published:** 1898-09

**Authors:** 


### Medical News and Miscellany.

---

**Dr. M. C. Bell** has changed his location from Wallis to San Angelo, Texas.

---

**Dr. J. C. Baird** has removed from Copperas Cove to Big Springs, Texas.

**Dr. N. P. Dolen** has removed from Houston to Fox, Texas.

---

**Dr. E. G. Condit** has removed from Salida, Colorado, to Aztec, New Mexico.

---

**Dr. Ambrose Morrison,** late professor of physiology in the Medical Department of the University of Nashville, died on August 3rd in the 52nd year of his age.

---

**Married**—In Austin, Texas, Aug. 18th, at the residence of the bride's father, Dr. P. M. Payne, of New Orleans, to Marie, eldest daughter of Dr. F. E. Daniel. Doctor and Mrs. Payne will make the City of Mexico their home.

---

**Dr. J. Shelton Horsely,** who for some years has been associated with Prof. Jno. A. Wyeth in his private and hospital work, has located in El Paso, Texas. Dr. Wyeth speaks very highly of Dr. Horsley, and the JOURNAL welcomes him to the ranks of the Texas profession.

---

**A Timely Book.**—We are in receipt of a cloth bound copy of a book issued by The Lambert Pharmacal Co., giving much valuable information on the subject of summer complaints or choleraic diarrhœa. It is a compilation of articles—or abstracts of articles on the subject by some of our ablest and best known clinicians. Messrs. Lambert Co., will mail a copy of their valuable work free to any reader of the Red Back who will ask for it.

---

**Sad Accident.**—A sweet little girl while playing "On the Banks of the Wabash" fell in the river and was drowned. Boo-hoo!

Another: A "loaded" bum fell on the sidewalk in front of the Blue Ruin Saloon. No damage done. He didn't "go off"; had to be carried off.

It is also reported that an explosion took place the other day at our great dam; the wind *blew up* the river!

The U. S. Government is buying up the entire pecan crop of Texas; wants the kernels for the army and the hulls for the navy.

[These are hot weather, war "*goaks*," as A. Ward would say.]

**Justice to the Journal of the American Medical Association.**—It is with extreme satisfaction that we call the attention of our readers to the denial and refutation by the *Journal of the American Medical Association* of the charge that it had accepted an advertisement of a popular nostrum, yclept "Ripans Tabules."—*American Journal Gynecology and Obstetrics*, New York.

The Red Back threw the proposition in the waste-basket.

